# Hypoalbuminemia is a frequent marker of increased mortality in cardiogenic shock

**DOI:** 10.1371/journal.pone.0217006

**Published:** 2019-05-16

**Authors:** Toni Jäntti, Tuukka Tarvasmäki, Veli-Pekka Harjola, John Parissis, Kari Pulkki, Tuija Javanainen, Heli Tolppanen, Raija Jurkko, Mari Hongisto, Anu Kataja, Alessandro Sionis, Jose Silva-Cardoso, Marek Banaszewski, Jindrich Spinar, Alexandre Mebazaa, Johan Lassus, for the CardShock investigators

**Affiliations:** 1 Cardiology, University of Helsinki and Department of Cardiology, Heart and Lung Center, Helsinki University Hospital, Helsinki, Finland; 2 Emergency Medicine, University of Helsinki and Department of Emergency Medicine and Services, Helsinki University Hospital, Helsinki, Finland; 3 Heart Failure Clinic and Secondary Cardiology Department, Attikon University Hospital, Athens, Greece; 4 Laboratory Division, Turku University Hospital and Department of Clinical Chemistry, University of Turku, Turku, Finland; 5 Intensive Cardiac Care Unit, Cardiology Department, Hospital de la Santa Creu i Sant Pau, Biomedical Research Institute IIB‐SantPau, Universidad Autónoma de Barcelona, Barcelona, Spain; 6 Department of Cardiology, CINTESIS, Porto Medical School, São João Hospital Center, University of Porto, Porto, Portugal; 7 Intensive Cardiac Therapy Clinic, Institute of Cardiology, Warsaw, Poland; 8 Department of Internal Medicine and Cardiology, University Hospital Brno, Brno, Czech Republic; 9 INSERM U942, University Paris Diderot and Department of Anesthesia and Critical Care, Hôpital Lariboisière, APHP, Paris, France; Erasmus Medical Center, NETHERLANDS

## Abstract

**Introduction:**

The prevalence of hypoalbuminemia, early changes of plasma albumin (P-Alb) levels, and their effects on mortality in cardiogenic shock are unknown.

**Materials and methods:**

P-Alb was measured from serial blood samples in 178 patients from a prospective multinational study on cardiogenic shock. The association of hypoalbuminemia with clinical characteristics and course of hospital stay including treatment and procedures was assessed. The primary outcome was all-cause 90-day mortality.

**Results:**

Hypoalbuminemia (P-Alb < 34g/L) was very frequent (75%) at baseline in patients with cardiogenic shock. Patients with hypoalbuminemia had higher mortality than patients with normal albumin levels (48% vs. 23%, p = 0.004). Odds ratio for death at 90 days was 2.4 [95% CI 1.5–4.1] per 10 g/L decrease in baseline P-Alb. The association with increased mortality remained independent in regression models adjusted for clinical risk scores developed for cardiogenic shock (CardShock score adjusted odds ratio 2.0 [95% CI 1.1–3.8], IABP-SHOCK II score adjusted odds ratio 2.5 [95%CI 1.2–5.0]) and variables associated with hypoalbuminemia at baseline (adjusted odds ratio 2.9 [95%CI 1.2–7.1]). In serial measurements, albumin levels decreased at a similar rate between 0h and 72h in both survivors and nonsurvivors (ΔP-Alb -4.6 g/L vs. 5.4 g/L, p = 0.5). While the decrease was higher for patients with normal P-Alb at baseline (p<0.001 compared to patients with hypoalbuminemia at baseline), the rate of albumin decrease was not associated with outcome.

**Conclusions:**

Hypoalbuminemia was a frequent finding early in cardiogenic shock, and P-Alb levels decreased during hospital stay. Low P-Alb at baseline was associated with mortality independently of other previously described risk factors. Thus, plasma albumin measurement should be part of the initial evaluation in patients with cardiogenic shock.

**Trial registration:**

NCT01374867 at ClinicalTrials.gov.

## Introduction

Hypoalbuminemia is a frequent finding both in chronic illness[1] and acute conditions[2]. In chronic illness hypoalbuminemia has been attributed to decreased albumin synthesis due to wasting and cachexia[3,4], although recent literature suggests that increased catabolism is more often the cause[5]. In acute conditions the mechanisms contributing to hypoalbuminemia differ from chronic disease as the major cause of hypoalbuminemia in the acute setting is capillary leakage into the interstitial space due to inflammatory processes[6]. In addition, decreased synthesis, haemodilution due to fluid administration, renal and gut losses due to congestion, and increased catabolism also play a role[5,7,8].

The association of hypoalbuminemia with increased mortality has been described in detail for end-stage renal disease[9] but it has also been established in varied conditions such as trauma[10], critical illness[7], cancer[11], chronic heart failure[12,13] as well as in the elderly[14]. More recently, the role of albumin has attracted attention also in acute cardiac conditions. Hypoalbuminemia has been shown to be associated with an increase in the rate of complications[15,16] and long-term mortality[16] in acute myocardial infarction, as well as worse outcomes in acute heart failure[17–19].

Cardiogenic shock is the most severe form of acute heart failure characterized by a low cardiac output resulting in low blood pressure and hypoperfusion[20]. The most common cause of cardiogenic shock is acute myocardial infarction[21]. Inflammatory and neurohormonal responses play a central role in the pathophysiology of cardiogenic shock[22], but the prevalence of hypoalbuminemia and its effect on mortality remains unexplored.

The purpose of this study was to investigate the prevalence and prognostic significance of plasma albumin (P-Alb) in patients with cardiogenic shock. Furthermore, we explored factors associated with hypoalbuminemia and changes in albumin levels during hospitalization.

## Materials and methods

The CardShock study (NCT01374867 at www.clinicaltrials.gov) is a European prospective, observational, multicentre and multinational study on cardiogenic shock. Recruitment was conducted between October 2010 and 31 December 2012. The study enrolled patients from emergency departments, cardiac and intensive care units, as well as catheter laboratories in nine tertiary hospitals from eight countries. The study was approved by the following ethics committees: Athens: Ethics Committee of Attikon University Hospital; Barcelona: Health Research Ethics Committee of the Hospital de Sant Pau; Brescia: Ethics Committee of the Province of Brescia; Brno: Ethic committee of University Hospital Brno; Helsinki: The Ethics Committee, Department of Medicine, The Hospital District of Helsinki and Uusimaa; Porto: Ethics committee of S. João Hospital Center/Porto Medical School; Rome: Ethical Committee Sant’Andrea Hospital; Warsaw: Local Bioethics Committee of the Institute of Cardiology. Copenhagen: The study was approved by the Danish Protection Agency with reference number GEH-2014-013; I-Suite number: 02731. The study was conducted in accordance with the Declaration of Helsinki. Written informed consent was obtained from the patient or next of kin if the patients were unable to give the consent on admission.

### Inclusion criteria and data collection

Consecutive patients aged over 18 years were enrolled in the study within 6 hours from identification of cardiogenic shock. The inclusion criteria were (1) an acute cardiac cause for the shock, and (2) systolic blood pressure <90mmHg (after adequate fluid challenge) for 30min or a need for vasopressor therapy to maintain systolic blood pressure >90mmHg, and (3) signs of hypoperfusion (any of the following: altered mental status, cold periphery, oliguria <0.5mL/kg/h for the previous 6 h, or blood lactate >2 mmol/L) (for details see Harjola et al.[21]). Exclusion criteria were shock caused by ongoing hemodynamically significant arrhythmia or shock after cardiac or non-cardiac surgery. The etiology of cardiogenic shock was determined by local investigators. Acute coronary syndrome etiology was defined as shock caused by myocardial infarction (with or without ST-elevation). Echocardiography was performed per protocol at study entry. Patients were treated according to local practice, and treatment and procedures were registered.

The study cohort consists of 178 patients with plasma samples available at baseline. Blood was drawn within 3 hours of study enrollment. Additionally, serial blood samples were collected at 12 h, 24 h, 36 h, 48 h and 72h (all +/-3 h). Separated plasma was immediately frozen in aliquots and stored at −80°C. Creatinine, C-reactive protein (CRP), high-sensitivity troponin T (hsTnT), N-terminal pro b-type natriuretic peptide (NT-proBNP), alanine aminotransferase, alkaline phosphatase, total bilirubin, and albumin (P-Alb) (Roche Diagnostics, Basel, Switzerland) were analyzed from the plasma samples at a central accredited laboratory (ISLAB, Kuopio, Finland). The reference limit used for hypoalbuminemia was <34 g/L as recommended by the central laboratory, and has also been used in several studies on heart failure[12,13,18,23]. Arterial blood lactate and haemoglobin were analysed locally. Estimated glomerular filtration rate (eGFR) was calculated from creatinine values using the CKD-EPI (Chronic Kidney Disease Epidemiology Collaboration) equation[24]. Central venous pressure was recorded at 72 hours in 65 patients with a central venous line.

The primary endpoint was all-cause 90-day mortality. Vital status during follow-up was determined through direct contact with the patient or next of kin, or through population and hospital registers. Two patients were lost to follow-up. The study was approved by local ethics committees and conducted in accordance with the Declaration of Helsinki.

### Statistical analysis

Results are presented as numbers (n) and percentages (%), means and standard deviations (SD) for normally distributed variables, or median and interquartile range (IQR) for variables with a skewed distribution. Categorical variables were compared using Chi-squared or Fisher’s exact test whereas Mantel-Haenszel trend test was used for ordinal variables. Between-group comparisons were performed using two-way analysis of variance, Student’s t-test or Mann–Whitney U-test, as appropriate. Associations between continuous variables were assessed using Pearson and Spearman correlations for normally and non-normally distributed variables, respectively. Differences in survival between groups were assessed by Kaplan–Meier survival curves and log-rank test. The significance of changes in albumin levels between different time points was tested using a paired-samples T-test. Logistic regression analysis was used to identify variables associated with baseline hypoalbuminemia. To determine baseline variables independently associated with hypoalbuminemia, variables with a univariable p<0.10 were entered into a multivariable logistic regression model. For the selection of independently associated variables, both forward and backward conditional and likelihood ratio models were used. Receiver operating characteristics analysis was used to select the multivariable model with the highest area under the curve for predicting baseline hypoalbuminemia. Multivariable logistic regression models were used to test for the independent association between plasma albumin and 90-day mortality. Multivariable adjustments were made for 1) variables statistically significantly associated with hypoalbuminemia at baseline (p<0.05), i.e. smoking status, comorbidities (heart failure with reduced ejection fraction, coronary artery disease, prior myocardial infarction), calcium-channel blocker use, lung oedema on X-ray, BMI, eGFR, haemoglobin, NT-proBNP, and CRP at baseline, and presence of multi-vessel disease in primary coronary angiography, as well as 2) contemporary risk prediction models in cardiogenic shock such as the CardShock risk score[21], the IABP-SHOCK II score[25], and combinations of 1) and 2). To assess whether incorporating plasma albumin to the multivariable model provided incremental prognostic value, the likelihood ratio test for nested models was used. Discrimination was also assessed by integrated discrimination index (IDI) and clinical risk stratification by net reclassification improvement (NRI) [26]. Results from the regression analyses are presented as odds ratios (ORs) with 95% confidence intervals (CIs). A two-sided p-value <0.05 was regarded statistically significant. Data were analyzed using the SPSS statistical package, version 23 (IBM Corp, Armonk, NY) with the exception of the reclassification analyses which were performed with R version 3.5.1[27] using packages Hmisc and pROC.

## Results

### Patient characteristics

The mean age in the cohort was 66 years, and 26% were women. On average, mean arterial pressure at inclusion was 57 (SD 11) mmHg. The most common etiology of cardiogenic shock was acute coronary syndrome (ACS) (80%). The overall 90-day all-cause mortality was 42%. The mean baseline P-Alb was 29.5 g/L (SD 6.4 g/L, range 11–42 g/L). Hypoalbuminemia (P-Alb <34 g/l) at admission was observed in 75% (134/178) of patients. There was no difference in the P-Alb levels at baseline between ACS and non-ACS etiologies of cardiogenic shock (P-Alb 29.8 g/L vs. 28.3 g/L, p = 0.7)

### Characteristics of hypoalbuminemic cardiogenic shock patients

Baseline characteristics and clinical presentation of patients with and without hypoalbuminemia are shown in Table 1. Compared to patients with normal P-Alb levels, hypoalbuminemic patients were more likely to have a history of chronic diseases, such as prior myocardial infarction, ischemic heart disease, heart failure, and worse renal function. There were fewer current smokers and less use of calcium channel blockers in the hypoalbuminemic group. Notably, BMI was lower in the hypoalbuminemic group compared to the group with normal albumin levels.

**Table 1:** Patient characteristics and mortality in normoalbuminemic and hypoalbuminemic cardiogenic shock patients.

	All	Normoalbuminemia	Hypoalbuminemia	p-value
		(P-Alb ≥34g/L)	(P-Alb <34 g/L)	
**Demographics**	(n = 178)	(n = 44)	(n = 134)	
Age, years; mean (SD)	66 (12)	64 (12)	67 (12)	0.10
Smoking	72 (41)	23 (54)	49 (37)	0.05
Women	46 (26)	33 (29)	13 (20)	0.20
BMI, kg/m^2^; mean (SD)	27.0 (4)	28.2 (4)	26.6 (4)	0.03
**Medical history**				
Hypertension	108 (61)	29 (66)	79 (59)	0.41
Coronary artery disease	58 (33)	9 (21)	49 (37)	0.05
Previous myocardial infarction	45 (25)	5 (11)	40 (30)	0.01
Prior CABG	11 (6)	1 (2)	10 (8)	0.30
History of HFrEF	22 (13)	1 (2)	21 (16)	0.02
Diabetes mellitus	53 (30)	9 (21)	44 (33)	0.12
**Medications in use at admission**				
ACEI	53 (30)	15 (34)	37 (28)	0.51
ARB	26 (15)	6 (14)	20 (15)	0.81
Calcium-channel blockers	22 (12)	10 (23)	13 (10)	0.04
Beta-blocker	67 (38)	14 (32)	53 (40)	0.42
Diuretics	53 (31)	11 (26)	42 (32)	0.45
**Clinical presentation**				
Cold periphery	170 (96)	40 (93)	130 (97)	0.24
Confusion	117 (67)	29 (66)	88 (67)	0.93
Oliguria	94 (54)	24 (55)	70 (53)	0.90
Lactate > 2	125 (71)	29 (66)	96 (73)	0.35
ACS etiology	143 (80)	38 (86)	105 (78)	0.25
Lung oedema on X-ray	60 (36)	10 (23)	50 (40)	0.04
Systolic BP, mmHg; mean (SD)	77 (12)	76 (12)	79 (13)	0.52
Mean arterial pressure, mmHg; mean (SD)	57 (11)	58 (12)	57 (10)	0.43
Heart rate, beats/min; mean (SD)	88 (29)	87 (30)	89 (29)	0.77
LVEF, %; mean (SD)	33 (14)	35 (13)	32 (14)	0.09
Time from detection of shock to baseline, h:min; median (IQR)	2:00 (0:22–4:00)	2:00 (0:00–4:03)	2:00 (0:30–3:30)	0.86
**Mortality**				
90-day mortality	74 (42)	10 (23)	64 (48)	0.004

Results shown as n (%) for categorical and mean (SD) or median (IQR) for continuous variables. ACEI = angiotensin-converting enzyme inhibitor; ACS = acute coronary syndrome; ALT = alanine aminotransferase; ARB = angiotensin receptor blocker; BMI = body mass index; BP = blood pressure; CABG = coronary artery bypass grafting; HFrEF = heart failure with reduced ejection fraction; IQR = interquartile range; LVEF = left ventricular ejection fraction; NT-proBNP = N-terminal prohormone of B-type natriuretic peptide; SD = standard deviation

As shown in Table 2, there were no differences in lactate, mean arterial pressure, or systolic blood pressure between the groups. However, patients with hypoalbuminemia were more likely to have pulmonary oedema on chest X-ray, as well as higher levels of NT-proBNP and CRP, and lower levels of haemoglobin at baseline. There were no significant differences in liver function tests (alanine aminotransferase, alkaline phosphatase and total bilirubin) at baseline. In multivariable analysis, independent associates of hypoalbuminemia were higher CRP at baseline, pulmonary oedema on chest X-ray, history of heart failure with reduced ejection fraction, older age and calcium channel blocker use prior to admission (Table 3). Coronary angiography was performed in 136 patients, in which patients with hypoalbuminemia were more likely to have multi-vessel disease. Post-PCI, a higher proportion of patients with hypoalbuminemia had a TIMI grade flow of less than 3, but the difference was not statistically significant.

**Table 2:** Laboratory test results and angiographic findings in normoalbuminemic and hypoalbuminemic cardiogenic shock patients.

	All	Normoalbuminemia (P-Alb ≥34g/L)	Hypoalbuminemia (P-Alb <34 g/L)	p-value
**Laboratory test results at baseline**	(n = 178)	(n = 44)	(n = 134)	
eGFR, ml/min/1.73m2); mean (SD)	63 (30)	69 (26)	60 (30)	0.04
NT-proBNP, ng/L; median (IQR)	2710 (585–9434)	866 (226–5029)	3769 (1037–11745)	<0.001
CRP, mg/L; median (IQR)	16 (4–54)	7 (2–19)	25 (5–75)	<0.001
Leukocytes, 10E9; mean (SD)	14.0 (5.4)	14.7 (6.0)	13.8 (5.3)	0.30
Hemoglobin, g/L; mean (SD)	129 (23)	139 (20)	125 (23)	<0.001
Albumin (g/L), mean (SD)	29.5 (6.4)	37.2 (2.3)	27.0 (5.1)	
Lactate (mmol/L); median (IQR)	2.7 (1.7–5.8)	2.4 (1.5–5.1)	2.9 (1.7–5.9)	0.15
hsTnT (ng/L); median (IQR)	2260 (398–5418)	2601 (386–6940)	2108 (403–5362)	0.69
Alanine aminotransferase (IU/L); median (IQR)	44 (20–92)	42 (22–86)	45 (20–103)	0.90
Alkaline phosphatase (IU/L); median (IQR)	61 (49–81)	67 (53–91)	60 (47–78)	0.11
Total bilirubin (umol/L); median (IQR)	9.6 (5.7–15.4)	10.0 (5.6–16.1)	9.5 (5.7–15.2)	0.95
Change in albumin between baseline and 72h (ΔAlb 0-72h) (g/L); mean (SD)	-5.0 (6.4)	-10.2 (6.2)	-2.5(4.7)	<0.001
Fluid balance at 72h (ml); mean (SD)	1389 (4241)	1765 (4215)	692 (4116)	0.19
**Angiographic findings**	(n = 136)	(n = 36)	(n = 100)	
Multivessel disease; n (%)	93 (68)	18 (50)	75 (75)	0.006
TIMI flow <3 post PCI	37 (30)	6 (18)	31 (35)	0.06
PCI complications	44 (28)	12 (29)	32 (27)	0.77

Results shown as n (%) for categorical and mean (SD) or median (IQR) for continuous variables. CRP = C-reactive protein; eGFR = estimated glomerular filtration rate; IQR = interquartile range; NT-proBNP = N-terminal prohormone of B-type natriuretic peptide; PCI = percutaneous coronary intervention; SD = standard deviation; TIMI = thrombolysis in myocardial infarction

**Table 3:** Factors independently associated with hypoalbuminemia at baseline.

	Odds ratio	95% CI	p-value
CRP at baseline; mg/L	1.02	1.003–1.03	0.01
Lung oedema on chest X-ray	2.9	1.2–7.1	0.02
History of HFrEF	11.7	1.4–98	0.02
Age; years	1.04	1.01–1.07	0.02
Use of calcium-channel blocking medication	0.3	0.1–0.9	0.03

CI = confidence interval; HFrEF = geart failure with reduced ejection fraction

### Hypoalbuminemia and outcome

**Fig 1:**
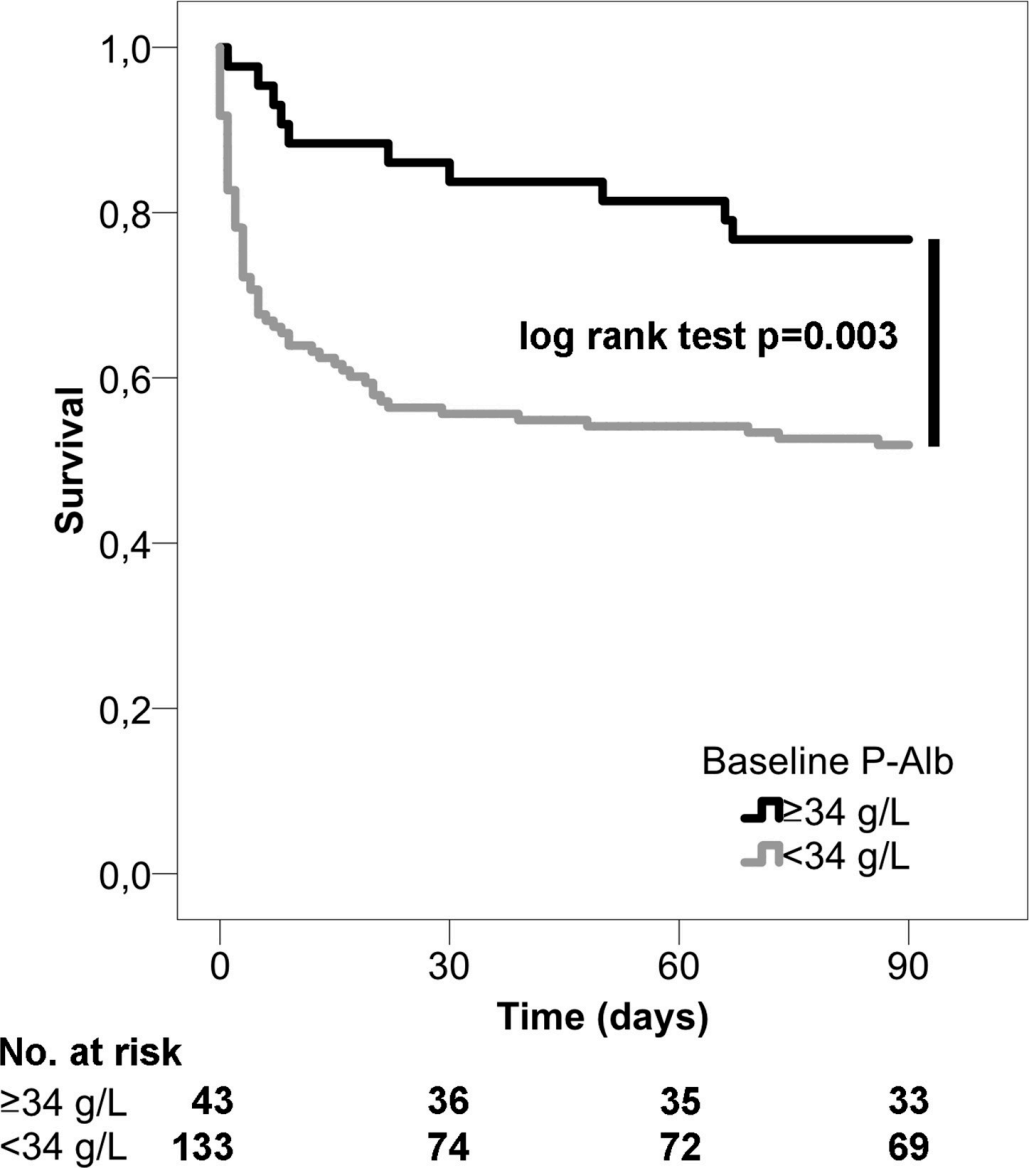
Kaplan-Meier survival curves of 90-day mortality according to baseline plasma albumin (P-Alb).

**Fig 2:**
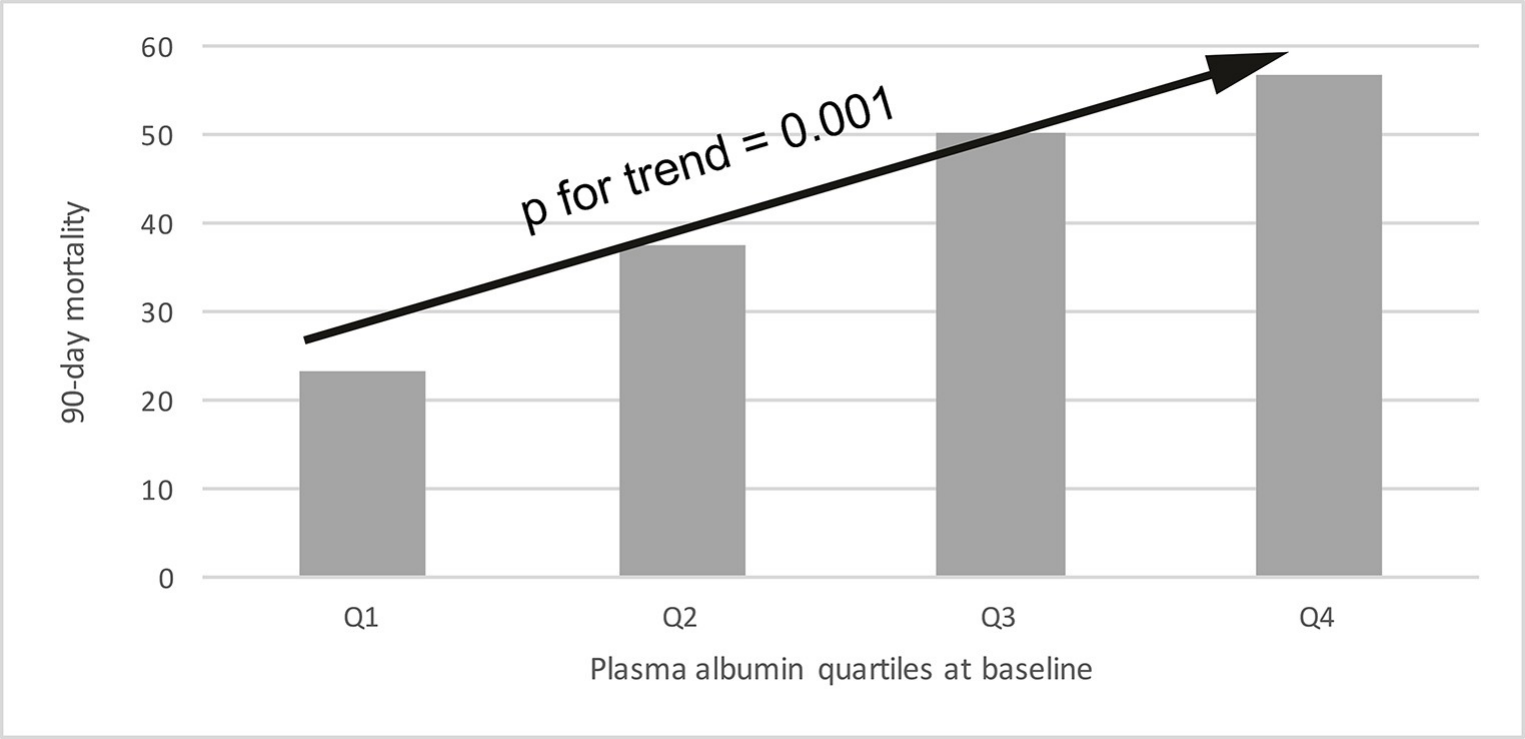
90-day mortality by baseline albumin quartiles. The P-Alb ranges for the quartiles were 34.0–42.9 g/L for the 1^st^ quartile, 30.0–33.9 g/L for the 2^nd^ quartile, 25.9–29.9 g/L for the 3^rd^ quartile and 10.4–25.9 g/L for the 4^th^ quartile.

Hypoalbuminemia at baseline was associated with a higher 90-day mortality compared to normal P-Alb levels (48% vs 23%, p = 0.004; Fig 1). Fig 2 shows that 90-day mortality increased across P-Alb quartiles from 23% in the highest quartile to 57% in the lowest quartile (p<0.001 for trend).

In unadjusted logistic regression analysis, baseline P-Alb had an OR of 2.4 per 10 g/L decrement (95% CI 1.5–4.1, p = 0.001). Multivariable adjustment did not significantly alter the results. Lower baseline albumin was associated with mortality independently of the CardShock risk score, the IABP-SHOCK II score and the variables associated with hypoalbuminemia (Table 4). Addition of P-Alb improved the risk prediction model compared with either the CardShock risk score or the IABP-SHOCK II score alone (χ2 = 5.301, p = 0.02 and χ2 = 7.088, p = 0.008 for comparison of nested models, respectively). Discrimination was also assessed using integrated discrimination index (IDI) and clinical risk stratification by net reclassification improvement (NRI) (Table 5).

**Table 4:** Unadjusted and adjusted odds ratios for baseline plasma albumin with 90-day mortality.

	Baseline plasma albumin per 10 g/L decrease	95% CI	p-value
Unadjusted	OR 2.4	1.5–4.1	0.001
Adjusted with variables associated with hypoalbuminemia^a^	OR 2.9	1.2–7.1	0.02
Adjusted with CSS score^b^	OR 2.0	1.1–3.8	0.03
Adjusted with CSS score^b^ and variables associated with hypoalbuminemia^a^	OR 2.9	1.02–8.4	0.045
Adjusted with IABP-SHOCK II score^c^	OR 2.5	1.2–5.0	0.01
Adjusted with IABP-SHOCK II score^c^ and variables associated with hypoalbuminemia^a^	OR 7.4	1.7–31.3	0.007

OR = odds ratio; CI = confidence interval

^a^Comorbidities (heart failure with reduced ejection fraction, ischaemic heart disease), smoking status, calcium-channel blocker use, lung oedema on X-ray, body mass index, haemoglobin, NT-proBNP and CRP at baseline, presence of multi-vessel disease in primary coronary angiography

^b^age >75 years (1 point), history of myocardial infarction or coronary bypass (1 point), altered mental status at presentation (1 point), ACS etiology (1 point), left ventricular ejection fraction <40% (1 point), lactate (2–4 mmol/l = 1 point, >4 mmol/l = 2 points) and estimated glomerular filtration rate on admission (60–30 mL/min/1.73 m2 = 1 point, <30 mL/min/1.73 m2 = 2 points)

^c^Age >73 years (1 point), history of stroke (1 point), blood glucose >10.6 g/L at baseline (1 point), creatinine >132.6 umol/L at baseline (1 point), TIMI flow <3 post-PCI (2 points), blood lactate >5 mmol/L (2 points)

**Table 5:** Comparison of cardiogenic shock risk score models.

Model	AUC (95% CI)	Continuous NRI (95% CI)	IDI (95% CI)
CardShock risk score	0.798 (0.734–0.862)		
CardShock risk score + P-Alb	0.819 (0.757–0.881)	0.297 (-0.006–0.600)	0.027 (0.003–0.051)
IABP II SHOCK -score	0.719 (0.629–0.808)		
IABP II SHOCK -score + P-Alb	0.750 (0.661–0.839)	0.355 (-0.004–0.715)	0.054 (0.013–0.095)

AUC = area under curve; CI = confidence interval; IDI = integrated discrimination index; NRI = net reclassification index; P-Alb = baseline plasma albumin

### Serial P-Alb measurements and changes during hospitalization

**Fig 3:**
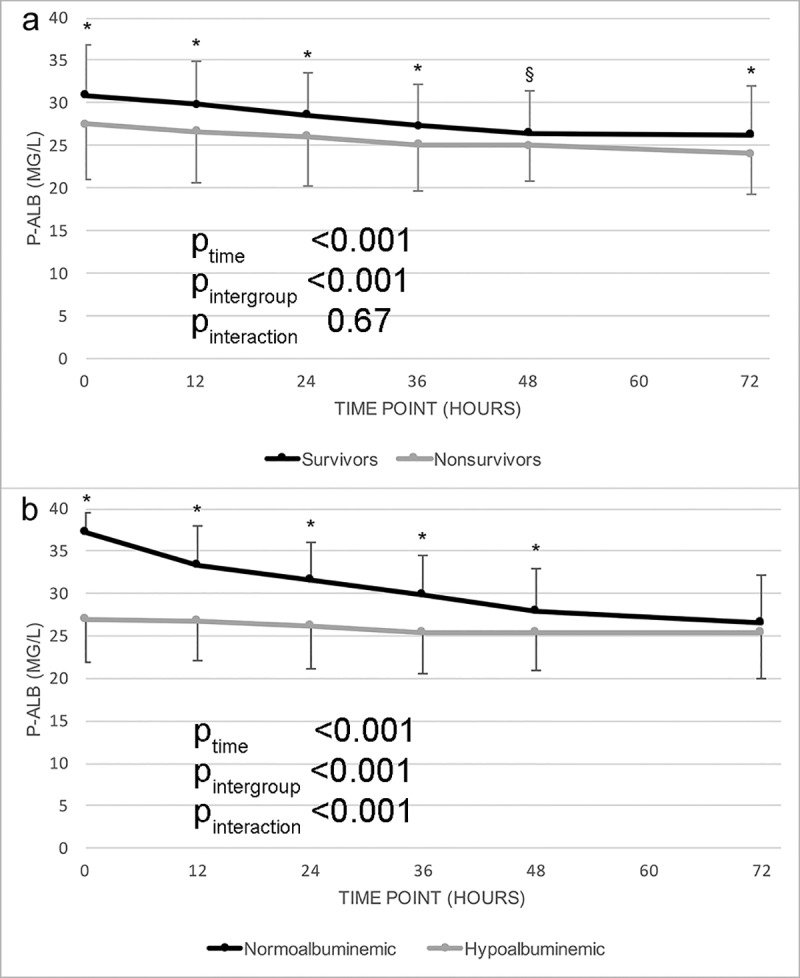
**A: Mean plasma albumin at different time points during hospitalization in 90-day survivors and non-survivors of cardiogenic shock.** Mean change between 0 and 72h -4.6 g/L for survivors, -5.4 g/L for non-survivors; p = 0.54. **B: Plasma albumin at different time points during hospitalization in patients with normoalbuminemia or hypoalbuminemia at baseline.** Mean change between 0 and 72 h -10.8 mg/L for normoalbuminemic patients and -2.5 mg/L for hypoalbuminemic patients; p<0.001. P-values in the picture represent results for linear mixed model analysis of variance for repeated measures. * p<0.05 § p<0.10 for the difference in P-Alb between groups at this time point (Student’s t-test). Error bar = standard deviation.

Plasma albumin concentrations decreased during hospitalization, on average -5.0 g/L during the first 72 hours (ΔAlb0-72h). Albumin levels decreased similarly in survivors and non-survivors (-4.6 g/L vs. 5.4 g/L, p = 0.5, Fig 3A). The P-Alb levels were lower for non-survivors throughout the follow-up period of 72 hours compared to 90-day survivors. The downward trend in P-Alb from baseline until 72h was statistically significant for both survivors and non-survivors (p<0.001 for both). P-Alb decreased more rapidly among patients with normal P-Alb at baseline compared with hypoalbuminemic patients (Table 2, Fig 3B). However, P-Alb decrease (ΔAlb0-72h) was not associated with mortality, even after adjustment for baseline P-Alb (OR 1.0, 95% CI 0.94–1.06, p = 0.87, adjusted for baseline albumin OR 0.94, 95% CI 0.87–1.02, p = 0.17). At 72 h, ΔAlb0-72h correlated negatively with fluid balance (Pearson correlation coefficient (r_p_) = -0.41, p<0.001) and with CRP (Spearman correlation coefficient (r_s_) = -0.41, p<0.001), but positively with alkaline phosphatase (r_s_ = 0.28, p = 0.002) and total bilirubin (r_s_ = 0.26, p = 0.005). The negative correlation with central venous pressure at 72h had borderline significance (r_p_ = -0,26, p = 0.051).

## Discussion

This is the first study on the prevalence and prognostic value of P-Alb in cardiogenic shock. First, low P-Alb was a frequent finding (75%) early in cardiogenic shock, with most of the patients having values below 30 g/L already at baseline. Second, hypoalbuminemia was associated with higher mortality independent of other variables. Third, P-Alb levels decreased during hospitalization in all patients, but the rate of change was not associated with 90-day mortality. Nevertheless, plasma albumin levels were lower in non-survivors compared to survivors during the duration of the study period.

The high proportion of hypoalbuminemic patients in this study was striking, and probably has various causes. First, the association of hypoalbuminemia with comorbidities suggests that hypoalbuminemia may be a pre-existing condition, perhaps linked with frailty and nutritional status, as the lower hemoglobin levels and BMI in the hypoalbuminemic group could suggest. Second, it has been suggested that hypoalbuminemia might predispose STEMI patients to cardiogenic shock[16], which would lead to a higher prevalence of hypoalbuminemia in cardiogenic shock populations (75% in our study compared to 30% in STEMI[16]). Furthermore, inflammatory response and SIRS in cardiogenic shock[28] increase capillary permeability promoting the transcapillary escape rate of albumin[6]. Interestingly, hypoalbuminemia did not appear to be linked to the severity of cardiogenic shock, as for example lactate or blood pressure levels did not differ between normo- and hypoalbuminemic patients.

Hypoalbuminemia has been shown to be associated with worse outcomes in acute coronary syndromes[15,16], acute heart failure[17,18] and critical illness[7,29,30]. We show that in cardiogenic shock mortality increases in a linear fashion with decreasing baseline P-Alb levels. Patients with normal albumin levels at baseline had a relatively favourable outcome, whereas moderate or severe hypoalbuminemia was associated with two-fold higher mortality. In a meta-analysis of hypoalbuminemia in acutely ill patients, it was estimated that each 10 g/L decrease in serum albumin concentration increased the odds of mortality by 137%[2]. In line with this estimation the unadjusted odds of 90-day mortality increased by 140% for each 10g/l decrement in our study, and lower albumin levels were significantly associated with mortality in analyses adjusted for multiple covariates.

The independent association of hypoalbuminemia with mortality suggests that hypoalbuminemia may have effects on mortality which are not explained by other variables interacting with hypoalbuminemia. Oduncu et al. suggested that hypoalbuminemia may play a direct role in poor reperfusion after PCI[16]. Interestingly in this respect, albumin has been suggested to have anticoagulative properties[29]. It has also been implied that albumin may be associated with disease severity instead of just the presence or absence of disease, in which case categorizing pre-existing diseases as binary variables may lead to attributing the risk caused by disease severity to albumin[1]. In our study, there was a trend for TIMI flow <3 after primary PCI in hypoalbuminemic patients, however, the presence of multi-vessel coronary artery disease did not interfere with the independent association of P-Alb with mortality.

There are several possible pathways to explain the association between hypoalbuminemia, cardiogenic shock, outcome and the laboratory parameters associated with hypoalbuminemia in this study, such as lower hemoglobin and higher CRP-values, which may act in concert. One possible explanation could be aggressive fluid resuscitation prior to study enrollment and congestion leading to worse outcomes. Unfortunately, we do not have available data on fluid resuscitation prior to study enrollment to assess this possibility, but after study enrollment the fluid balance between normo- and hypoalbuminemic patients did not differ. Another possible pathway could be pre-existing chronic illness resulting in low-grade inflammation raising CRP, which would lead to anemia of chronic illness and hypoalbuminemia[31]. Also, infection or higher levels of inflammation reflected by the higher CRP-levels in hypoalbuminemic patients could lead to capillary leakage of albumin resulting in hypoalbuminemia[6].

In this study, there was no association between the rate of decrease in P-Alb during the first 72 hours and 90-day mortality. The significance of changes in albumin has been explored in ICU patients, but data are conflicting [7, 28]. We found that the rate of decrease of P-Alb was associated with the baseline level and differed between normo- and hypoalbuminemic patients. Correlations between ΔAlb0-72h and CRP, Bilirubin and AFOS at 72 hours also suggest that inflammation and cholestatic liver injury may play a role in the rate of decrease of P-Alb in cardiogenic shock.

The high prevalence of hypoalbuminemia and its independent association with outcome suggests that measuring P-Alb levels early in cardiogenic shock should be incorporated in clinical practice. As can be seen in Fig 3A, low P-Alb at later time points was also associated with mortality, but the association was strongest for early albumin levels (0-12h). As discussed above, albumin levels are subject to change due to various reasons and the rate of change did not predict mortality. As intravenous use of albumin has been shown not to decrease mortality in the critically ill [32], further studies are needed to determine if there are any other therapeutic options that would specifically target the worse prognosis associated with hypoalbuminemia in cardiogenic shock.

Our study has some limitations. First, it was not possible to have data on the patients’ albumin levels before study enrollment to determine whether the observed hypoalbuminemia was pre-existing or not. Second, we did not have information on the fluid status prior to study enrollment, as haemodilution due to excessive fluid resuscitation could be one of the causes of hypoalbuminemia. Third, although adjustments were made for various variables found to associate with hypoalbuminemia, there may be confounding factors we were unable to account for, leading to an overestimation of the independent association of P-Alb with mortality. However, the estimated ORs are in accordance with previous studies on the effect of hypoalbuminemia on mortality and the results were consistent in multiple analyses.

## Conclusions

In conclusion, hypoalbuminemia was a very frequent finding in the early phase of cardiogenic shock. P-Alb at baseline was independently associated with 90-day all-cause mortality, with mortality increasing across lower albumin quartiles. This study found that P-Alb is a prognostic marker in cardiogenic shock, and we suggest incorporating P-Alb measurement as part of the assessment of patients with cardiogenic shock.

## Supporting information

S1 Table Causes of death as reported by local investigators. (DOCX)

S1 Dataset De-identified patient data used in the study. (XLSX)
