# Supplementary material for: Hypoalbuminemia is a frequent marker of increased mortality in cardiogenic shock
Source: PLoS One. 2019 May 16;14(5):e0217006. doi: 10.1371/journal.pone.0217006 (PMC6522037; doi:10.1371/journal.pone.0217006)
Supplement: S1 Table — (DOCX) [file pone.0217006.s001.docx]

|  |  |
| --- | --- |
| Myocardial infarction | 43 (58%) |
| Worsening heart failure | 25 (34%) |
| Pulmonary embolism | 1 (1%) |
| Witnessed arrhythmic (VT/VF) sudden death | 3 (4%) |
| Stroke | 3 (4%) |
| Infection | 10 (14%) |
| Renal failure | 8 (11%) |
| Other cause | 12 (16%) |

Table S1. Causes of death as reported by local investigators. Note: more than 1 cause of death per patient was accepted.
